# Computer- and Smart-Tablet-Based Self-Administered Treatments in Chronic Post-Stroke Aphasia: A Systematic Review

**DOI:** 10.3390/brainsci15020122

**Published:** 2025-01-26

**Authors:** Célia Ericson, Alisa Latysheva, Sarah-Ève Poirier, Marion Fossard

**Affiliations:** 1 Institute of Logopedic Sciences, University of Neuchâtel, rue de la Pierre-à-Mazel 7, 2000 Neuchâtel, Switzerland; 2 Clinic La Lignière, La Lignière 5, 1196 Gland, Switzerland; alisa.latysheva@gmail.com; 3 Center for Interdisciplinary Research in Rehabilitation and Social Integration (Cirris), CIUSSS de la Capitale-Nationale, Quebec, QC G1M 2S8, Canada; sarah-eve.poirier.1@ulaval.ca; 4 School of Rehabilitation Sciences, Faculty of Medicine, Laval University, Quebec, QC G1V 0A6, Canada

**Keywords:** chronic post-stroke aphasia, language therapy, self-administered treatment, computer-based, smart-tablet-based

## Abstract

Background: In current clinical practice, resources remain limited and are insufficient to address the needs of people with chronic post-stroke aphasia. To improve access to speech therapy, self-administered therapies using computers or smart tablets are increasingly recommended. In addition to enabling more intensive and prolonged treatment, computer- and smart-tablet-delivered therapies can be highly enjoyable and motivating for patients. Aims: This systematic review aimed to identify computer- and smart-tablet-based self-administered treatments and analyze the proposed interventions in terms of treatment targets, effectiveness (considering specificity, generalization, transfer, and maintenance), and clinician involvement (during and/or prior self-administered therapies). Methods: Terms encompassing three main concepts (rehabilitation, self-administration, and aphasia) were used to search three electronic databases (Scopus, PubMed, and PsycINFO). Two reviewers independently screened titles and abstracts against eligibility criteria. Data extraction of included studies was completed by three reviewers. Results: Thirty-nine studies were included in this study. In terms of treatment targets, anomia is the most treated symptom in published studies (*n* = 24), but the existence of promising studies for other disorders means that the targets can be broadened. Therapies are effective for trained items, and gains are maintained. There is some evidence of transfer effects for treatments targeting the sentence level. Most studies offer training sessions, previous self-administered therapy, and/or observation and monitoring sessions during therapy; more rarely, self-administered therapy is supplemented with face-to-face therapy. Conclusions: This systematic review is the first to focus specifically on self-administered technology-based therapies. It provides important evidence-based information for clinical practice in self-administered therapies via computer or smart tablet.

## 1. Introduction

Even though the overall ischemic stroke incidence rate has decreased during the first decades of the 21st century, the number of patients living with the long-term effects of stroke is increasing and projected to grow in the coming decades due to population aging and reduced stroke mortality [1,2]. More than one-third of stroke survivors suffer from acquired aphasia, a language disorder that can affect all four language modalities (oral production, auditory comprehension, reading, and writing), and for many individuals, this condition persists well beyond the acute phase [1,2,3]. Aphasia is associated with higher mortality, disability, and use of health services [4,5] so it remains an important symptom that needs to be considered in stroke care and rehabilitation.

Speech and language therapy (SLT) is provided for people with aphasia (PWA), focusing on improving their language and communication abilities, and helping them to participate in daily communication activities. PWA often receive SLT in the initial months following a stroke, but many discontinue therapy or receive only minimal support during the chronic phase, despite evidence supporting its benefits [6]. This gap in care is primarily due to limited and insufficient clinical resources to meet the needs of PWA [7].

Several authors have shown that the effectiveness of SLT is strongly influenced by the dosage, with the benefits of higher-intensity approaches linked to greatest overall language and functional communication gains [8,9]. A recent review concluded that the most significant improvements in language and functional communication occurred with frequent SLT (5 days/week) and were augmented when supplemented by prescribed home practice [9]. Nonetheless, providing frequent SLT during the chronic phase is often impractical due to limited access to therapists, transportation difficulties, distant geography, scheduling constraints, fatigue, and, in some countries, due to limited insurance coverage [10]. Consequently, rehabilitation for chronic PWA is often insufficient.

One approach that meets intensity requirements and supports long-term treatment is self-administered home therapy, allowing PWA to engage in therapy tasks independently. In a data analysis of over 3500 users of a therapy platform for PWA, Godlove et al. [11] compared therapy delivered at home to therapy delivered only in clinic, and found that home therapy users received more frequent sessions (at least every 2 days) and completed tasks in less time than clinic patients, who received less frequent therapy (only once every 5 days).

In addition to intensifying treatment, self-administered remote therapies for PWA offer several advantages over face-to-face therapy. They allow patients to practice in a familiar environment and eliminate the need to travel between the clinic and home, making it easier for those in rural areas or with physical limitations to continue their SLT. Breaking sessions into multiple parts throughout the day helps reduce fatigue [12]. In addition, self-administered therapies could be a lower-cost option [13,14], particularly for PWA with mild or moderate difficulties [15], potentially halving the costs [16]. From the therapists’ perspective, self-administered therapies can reduce waiting lists [17], alleviate the workload, and increase the number of patients treated [18].

A scoping review focusing on self-management approaches for PWA revealed that technology-based interventions are predominantly used, enhancing autonomy and intensifying treatment [19]. In this approach, therapy is delivered on various computerized platforms, most commonly computers (51.1%) and smart tablets (40.4%), and follows the same principles as traditional face-to-face rehabilitation [20]. Computer-based tasks can be customized to suit PWA’s individual needs, considering their personal context and language ability levels. This personalization can help motivate practice [21], and the resulting autonomy and opportunities are attractive to PWA [22]. Currently, the rapid development and increasing accessibility of modern technologies have led to the expansion of therapies using digital platforms in aphasia rehabilitation. There are now numerous software offering therapy activities for PWA [23,24], mostly focusing on word retrieval or a combination of language skills [23,25].

In addition to patient compliance, technology-based interventions have proven efficacy and effectiveness for chronic aphasia rehabilitation [26,27,28]. There is also evidence that computer-delivered therapy is effective compared to no therapy and is as effective as clinician-delivered therapy [11,29,30]. While some interventions include direct clinician involvement during therapy sessions, others allow patients to practice independently at home, suggesting a flexible approach to integrating technology in both supervised and unsupervised settings [26]. A recent randomized controlled trial compared treatment outcomes for PWA receiving self-administered computerized word-finding therapy relative to other control treatments [16]. Results showed that self-administered computerized SLT improved word-finding more than usual care did. However, there was no evidence of generalization to untrained items, functional communication, or participant perception of their own communication or participation. Another study compared the effectiveness of a self-administered speech, language, and cognitive therapy using two delivery methods: computerized or paper workbooks [10]. The results showed a greater improvement in the participant perception of quality of life and performance on language tasks in the computerized treatment group. The authors assumed that these results could be explained by the presence of immediate feedback and an automatic adaptation of the level of difficulty of the exercises for the computerized treatment group.

All these findings support the growing notion that self-administered computerized therapy can be an effective way to rehabilitate individuals with post-stroke aphasia. This approach offers a real alternative to facilitate home practice, maximize repetitive practice, and meet the dosage requirements of high-intensity SLT [25], while allowing PWA to take an active role in their rehabilitation.

Furthermore, speech–language pathologists (SLPs) show a strong interest in incorporating technology into their treatment plans. According to a questionnaire administered to SLPs in Australia and the Netherlands, 93% and 97% of respondents, respectively, expressed interest in using therapy applications with their patients [31]. In terms of the content of therapy applications, a key requirement identified by SLPs is that these tools should enable PWA to practice various language skills beyond the single word level, such as at the sentence or discourse level.

In the current clinical reality, where clinical resources are limited and insufficient to meet the needs of chronic post-stroke PWA, it is high time to consider this emerging field of computerized or smart tablet-based self-administered therapies for chronic PWA. The aims of this review are to present the treatment targets of self-administered interventions using computer or smart tablet, determine their effectiveness, and consider clinician involvement in these evolving treatments. By highlighting these issues, our aim is to provide clinicians with solid and relevant information, enabling them to make the best possible use of self-administered approaches using computers or smart tablet.

The specific aims of this systematic review are as follows:

(1) Identify the targets of computerized or smart tablet-based self-administered interventions.
(2) Investigate the effectiveness of these interventions in terms of specificity, generalization, transfer, and maintenance of targeted aphasic symptoms.
(3) Consider the extent to which clinicians are involved before and/or during the proposed interventions.

## 2. Materials and Methods

This systematic review was conducted following guidelines proposed by the Preferred Reporting Items for Systematic Reviews and Meta-Analyses (PRISMA, 2020) [32]. The review protocol was specified before the systematic review was conducted but was not registered.

### 2.1. Research Strategy and Eligibility Criteria

A comprehensive literature search was conducted using three electronic databases: SCOPUS, PubMed, and Psyc’INFO. We limited our search to studies published in English or French, with no limit for the publication years. Database searches were performed in February 2024 and were updated in August 2024. The keywords used in the search included terms related to rehabilitation, self-administration, and PWA. The following terms were entered, and the Boolean operators and truncators were adapted to each database consulted: (rehabilitati * OR readapt * OR reeducation OR training OR treatment OR therapy OR remediati * OR recovery) AND (“self-manage *” OR “self-administ *” OR “self-guided” OR “self-train *” OR “home practice” OR “home use” OR “home train *” OR remot *) AND aphasi *. The eligibility criteria shown in Table 1 were set.

Additional references that met the inclusion and exclusion criteria were searched in the reference lists of the selected papers and were added manually.

### 2.2. Study Selection

The whole screening procedure was conducted independently by two examiners while recording reasons for exclusion of ineligible studies. The first selection step was based on the titles and abstracts of each record yielded by the search after all duplicates had been removed manually. The two examiners determined whether the articles were appropriate according to the eligibility criteria presented above. After the first selection step, the full text of the remaining articles was read, and the same eligibility criteria were used. During both steps of the selection process, any disagreements were resolved by discussion until consensus was reached.

### 2.3. Data Extraction

Three researchers independently extracted relevant data from all accepted articles using a predefined set of data fields on an a priori developed Microsoft Excel software form. When the study involved multiple conditions, only the data from participants who met the eligibility criteria were reported. Conversely, in group studies, data from all participants were included. In 50% of the studies, the first author checked the data to ensure the inter-rater reliability. Disagreements were resolved by discussion until the authors agreed on the most appropriate solution.

## 3. Results

**Figure 1:**
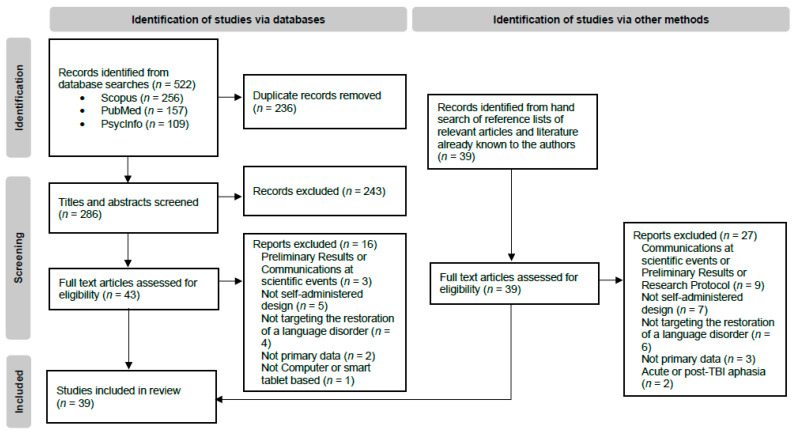
Flow diagram of the identification and selection records, adapted from the PRISMA flow diagram [32].

Results of the literature search are reported in a PRISMA flow diagram (Figure 1). The initial search yielded 522 records. After eliminating duplicates, the remaining 286 results were reviewed to ensure they met the inclusion criteria for title and abstract screening. Agreement for inclusion of titles and abstracts was >90%. Following this step, 43 full-text articles were assessed for eligibility. An additional 39 articles, identified through a manual search of the reference lists of relevant studies, were also evaluated for eligibility through full-text review. Agreement for inclusion of full texts was >90%. Finally, a total of 39 articles were obtained from the search procedure. Most of the excluded articles did not involve an intervention with a self-administered design (the frequency of self-administered sessions was not higher than the frequency of sessions in the presence of the clinician), or the article did not provide enough information to determine this.

### 3.1. Characteristics of the Included Studies

The descriptive characteristics of the included studies are shown in Table 2, organized by treatment targets. The following information is provided for each article: author(s) and publication year, language and the number (*n*) of participants, characteristics of interventions (software/program used; type of treatment) and efficacy measures (specificity; generalization; transfer; maintenance).

### 3.2. Targets of Computerized or Smart-Tablet-Based Self-Administered Therapies

The primary objective of this review is to identify the treatment targets that are addressed by self-administered therapies delivered via computers or smart tablets.

Five different treatment targets were identified in this review (see Table 2). Most of the studies focused on a single specific target: word production (24 studies: 61.5%), sentence processing and/or production (7 studies: 17.9%), reading (2 studies: 5.1%), or narrative production (1 study: 2.6%). Other studies covered more than one target (5 studies: 12.8%).

Word production: Of the 24 studies targeting word production, 22 focused on spoken-word production and two on written-word production [33,34]. Most studies targeted nouns, although some studies included verbs (*n* = 9) [33,34,35,36,37,38,39,40,41], proper nouns (*n* = 3) [14,42,43], or adjectives (*n* = 1) [41]. Words were most commonly selected from vocabulary databases (*n* = 15) [34,35,36,37,40,43,44,45,46,47,48,49,50,51,52], although some studies focused on personally relevant words, chosen by the PWA based on their interests or communication needs (*n* = 9) [12,14,33,38,39,41,42,53,54]. The therapies were delivered through a variety of programs or software (specialized or non-specialized). Specialized software, such as StepbyStep [14,38,39] or MossTalk Words [49,50], provided structured exercises with feedback and task adaptation. Non-specialized programs, such as Microsoft PowerPoint (e.g., [35,42]) or iBooks Author [36,37], presented words with varying levels of cueing through slides or interactive pages.

Sentence processing and/or production: Of the seven studies targeting sentence level, four trained personalized conversational scripts with diminishing cues, supported by a virtual SLP demonstrating correct speech movements (AphasiaScripts) [55,56,57,58]. Other treatments targeted production and comprehension of complex sentences (*n* = 1) [59], production of prepositions and sentences (*n* = 1) [60], or production of verbs and sentences (*n* = 1) [61].

Reading comprehension: Two studies targeted reading comprehension using an online adaptation of the Oral Reading for Language in Aphasia (ORLA) therapy [62,63]. The online system provided text and audio prompts, which mimicked SLP guidance and adjusted difficulty based on performance. One version included articulator movement models for the PWA to use as a reference [63].

Narrative production: One study targeted narrative production using an imitation-based therapy (IMITATE [64]), in which participants listened to and repeated words and phrases from different speakers.

Multiple domains: Among the five studies offering therapies targeting multiple domains, some programs focused exclusively on language tasks (*n* = 2), such as Tactus Therapy Solutions (reading, naming, comprehension, and writing) [65] and iAphasia (adding repetition and fluency) [66]. Others (*n* = 3) combined language and cognitive rehabilitation and provide access to around thirty different tasks (Constant Therapy) [10,67,68].

### 3.3. Effectiveness of Technology-Based Self-Administered Therapies

The second objective of this review was to investigate the effectiveness of these therapies for each targeted aphasic symptom. To achieve this, intervention outcomes were reported in terms of specificity (improvements on trained items), generalization (improvements on untrained items and/or standardized tests), transfer (improvements on connected speech or perceived change by PWA and/or significant other), and maintenance. None of the studies evaluated all the effectiveness measures.

**Figure 2:**
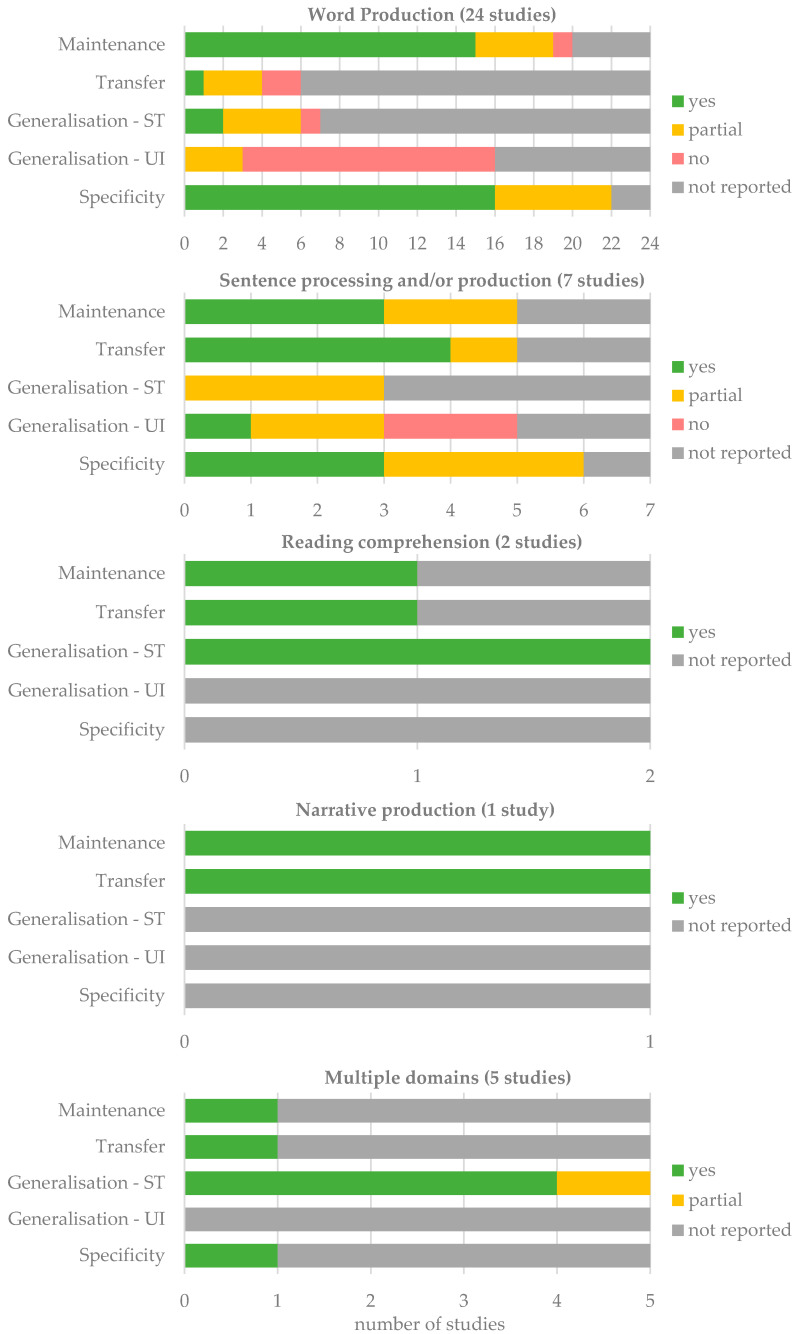
Summary of treatment effectiveness for targeted aphasic symptoms. Improvements are highlighted as follows: green for measures showing improvement in all groups or PWA, yellow for partial improvements (some groups or PWA), red for no improvement, and gray for unreported measures. UI, untrained items; ST, standardized tests.

Although there is considerable variability in the designs and objectives, all the selected studies unanimously highlighted the potential of self-administered, technology-based therapies to effectively reduce aphasic symptoms. Detailed data are presented in Table 2, while Figure 2 provides a summary of the effectiveness of these therapies for each targeted aphasic symptom.

When studies compared groups (e.g., self-administered vs. clinician-delivered therapy), only the results for the self-administered therapy group were reported. For information, these comparisons concerned five studies (two on word production [47,49], two on reading comprehension [62,63], and one on sentence production [59]). These studies concluded that both approaches yielded comparable outcomes in terms of specificity and generalization patterns. Similarly, two randomized controlled trials on word production treatment reported significant improvements with self-administered computer-based therapy compared to usual care [39] and an attention control group (paper-based puzzle book activities) [14].

**Table 2:** Treatment target, descriptive characteristics, and treatment effect of the included studies.

Treatment Target	Author(s), Publication Year	Participants		Characteristics of Interventions: Treatment Type—Software/Program	Efficacy Measures				
		Language	n		Specificity	Generalization		Transfer	Maintenance
						Untrained Items	Standardized Tests		
Word production	Quique et al., 2022 [44]	Japanese	2	Cued naming with flashcards—Anki	2/2 *	0/2			2/2
	Gallée et al., 2020 [48]	English	6	Semantic and phonological naming—Constant Therapy			BNT: 4/6		
	Kurland et al., 2014 [36]	English	5	Cued naming—designed on iBooks Author	4/5	0/5			
	Kurland et al., 2018 [37]	English	21	Cued naming—designed on iBooks Author	Yes	No			Yes
	Lavoie et al., 2019 [12]	French	4	Semantic and phonological naming—iTSA	4/4	2/4		2/4	4/4
	Lavoie et al., 2016 [34]	French	1	Cued naming—designed on Keynote	1/1	0/1			1/1
	Heide et al., 2023 [41]	German	2	Cued naming—LingoTalk	2/2	0/2	WPP: 0/2	0/2	2/2
								#1/2	
	Choe et al., 2007 [47]	English	4	Cued naming—designed on Microsoft PowerPoint	2/4	0/4			3/4
	Choe et al., 2010 [42]	English	2	Cued naming—designed on Microsoft PowerPoint	2/2				1/2
	Choe and Stanton, 2011 [53]	English	2	Cued naming—designed on Microsoft PowerPoint	2/2	0/2			2/2
	Routhier et al., 2016 [35]	French	2	Cued naming—designed on Microsoft PowerPoint	2/2	0/2			2/2
	Mason et al., 2011 [54]	English	3	Picture-assisted repetition—designed on Microsoft PowerPoint	2/3	0/3		1/3	2/3
	Ball et al., 2011 [33]	English	3	Copy and recall therapy with repetition—Modified CART	3/3		naming WAB-R: 1/3		2/2
	Fink et al., 2002 [49]	English	3	Cued naming—MossTalk Words	3/3	0/3	PRT: 0/3; PORT: 2/3		3/3
	Ramsberger and Marie, 2007 [50]	English	4	Cued naming—MossTalk Words	4/4	2/4			4/4
	Fridriksson et al., 2009 [45]	English	10	Picture/word matching—NA	1/2 group		PNT: 1/2 group		
	Laganaro et al., 2003 [46]	French	3	Metaphonological therapy—NA	3/3	0/3			3/3
	Pedersen et al., 2001 [51]	Danish	3	Semantic, phonemic and written naming—NA	2/3				2/3
	Choe et al., 2023 [43]	English	1	Spoken-naming and upper-limb—NA	1/1				0/1
	Herbert et al., 2012 [52]	English	6	Syntactic cueing—STAR	5/6	0/6		4/6	Yes
	Mortley et al., 2004 [38]	English	7	Semantic, spelling, word-to-picture matching, naming—StepbyStep	7/7	3/7		#6/6	
	Palmer et al., 2012 [39]	English	16	Listening, cued naming—StepbyStep			OANB: Yes		Yes
	Palmer et al., 2019 [14]	English	83	Listening, cued naming—StepbyStep	Yes	No		No	Yes
	Trevittaya and Chinchai, 2023 [40]	Thai	5	Cued naming—Thai Naming	5/5		Thai WAB: 5/5		5/5
Sentence processing/production	Cherney and Halper, 2008 [57]	English	3	Script training, reducing cues—AphasiaScripts	2/3		WAB-AQ: 1/3; CETI: 1/2; CADL: 0/3	#2/3	1/3
	Manheim et al., 2009 [58]	English	20	Script training, reducing cues—AphasiaScripts				#Yes	Yes
	Cherney et al., 2014 [56]	English	8	Script training, reducing cues—AphasiaScripts	Yes	No			Yes
	Cherney et al., 2019 [55]	English	20	Script training, reducing cues—AphasiaScripts	Yes	No		Yes	Yes
	Hickin et al., 2023 [61]	English	6	Verb and sentence therapy—designed on Microsoft PowerPoint	5/6	2/6	CETI: 4/6		2/3
	Linebarger et al., 2001 [60]	English	3	Prepositions training—Prolog and C++	2/3			1/1	
	Thompson et al., 2010 [59]	English	6	Treatment of Underlying Forms—Sentactics	6/6	6/6	SPPT: 6/6; SCT: 0/6	Yes	
Reading comprehension	Cherney, 2010 [62]	English	11	Reading sentences—Web-ORLA			WAB-AQ: Yes	Yes	
	Cherney et al., 2021 [63]	English	19	Repeating and reading sentences—Web-ORLA			WAB-R LQ: Yes		Yes
Narrative production	Duncan and Small, 2017 [64]	English	18	Intensive imitation practice—IMITATE				Yes	Yes
Multiple domains	Kiran et al., 2014 [68]	English	3	Cognitive and language tasks—Constant Therapy			WAB-R, CLQT: 3/3; BNT: 2/3		
	Des Roches et al., 2015 [67]	English	42	Cognitive and language tasks—Constant Therapy	Yes		WAB-R, CLQT: Yes		
	Braley et al., 2021 [10]	English	17	Cognitive and language tasks—Constant Therapy			WAB-R AQ: Yes		
	Choi et al., 2016 [66]	Korean	8	Language tasks—iAphasia			K-WAB: Yes		Yes
	Stark and Warburton, 2016 [65]	English	7	Language tasks—Tactus Therapy Solutions			CAT: Yes	Yes	

Note. * These fractions refer to the number of PWA who showed improvements compared to the number of PWA for whom the effectiveness measure was conducted. Binary metrics (yes/no) are reported for the outcomes of group studies. Cells are highlighted in gray if the measure was not used. When the transfer measure was preceded by a #, it refers to a perceptive measure of change by PWA and/or significant other. Data from PWA which did not meet the inclusion criteria were not reported; BNT, Boston Naming Test [69]; CADL, Communication activities of daily living [70]; CAT, Comprehensive Aphasia Test [71]; CETI, Communicative Effectiveness Index [72]; CLQT, Cognitive Linguistic Quick Test [73]; K-WAB, Korean version of the Western Aphasia Battery [74]; NAVS, Northwestern Assessment of Verbs and Sentences [75]; OANB, Object and Action Naming Battery [76]; PORT, Philadelphia Oral Reading Test (part of PNT); PNT, Philadelphia Naming Test [77]; PRT, Philadelphia Repetition Test [78]; SCT, Sentence Comprehension Test of the NAVS; SPPT, Sentence Production Priming Test of the NAVS; Thai WAB [79]; WAB, Western Aphasia Battery [80]; WAB-R, Western Aphasia Battery-Revised [81]; AQ, Aphasia Quotient, LQ, Language Quotient; WPP, Wortproduktionsprüfung [82].

### 3.4. Clinician Involvement Before and/or During Proposed Interventions

The third objective of this review was to consider the extent of clinician involvement before and/or during the proposed interventions. The clinician’s involvement in treatment varied to some extent across studies (see Table 3). Involvement was categorized according to whether it occurred before or during the self-administered treatment period. Of note, one study did not report the SLP involvement [40].

Prior to self-administered treatment: A large majority of studies (24/39: 61.54%) reported clinician-led introduction and training sessions just before the start of the self-administered therapy. During these sessions, the clinician provided instructions and demonstrations on how to use the computer or smart tablet. In some cases, PWA were provided with a paper guide that outlined each step of the treatment.

In a few studies (4/39: 10.26%), an SLP-led therapy preceded the self-administered treatment, with results showing that home practice programs effectively promoted continued language recovery [36]. In three of these studies, the tasks used during the SLP-led therapy differed from those used in the self-administered therapy (although the treatment goals were similar) [36,37,46], while in one study, the tasks were identical [54].

During self-administered treatment: In most studies (21/39: 53.85%), the clinician intervened weekly or even in every session during the self-administered treatment—either in person or remotely—to observe and/or monitor the therapy progress without providing any cues or reinforcement. During these sessions, the clinician initiated the administration of the protocols, adjusted the treatment plan and the difficulty of the stimuli according to the PWA’s performance, guaranteed smooth computer operation, ensured appropriate and intensive practice, and/or recorded the participant’s adherence. Some self-administered therapies did not require clinician monitoring; they used self-learning applications that automatically adjusted the difficulty level based on PWA performance [38,65].

In some studies (6/39: 15.38%), weekly SLP-led therapy was conducted in parallel with self-administered therapy. These sessions provided regular supervision, reinforcement, and feedback on tasks identical to those completed during self-administered therapy. In two studies, PWA also attended weekly group sessions aimed at improving their communication skills in functional settings [42,47].

## 4. Discussion

Acknowledging the limited and insufficient clinical resources available to meet the needs of chronic post-stroke PWA, we questioned whether self-administered computer- or smart-tablet-based therapies could effectively support SLT. Thus, the goals of this systematic review were to identify treatment targets of self-administered computer or tablet-based therapies, to assess the immediate and long-term effectiveness of these treatments as well as their transfer (to connected speech or perceived change by PWA and/or significant other), and to investigate the clinician involvement in administering such therapies for chronic post-stroke aphasia. Based on predefined selection criteria and inter-rater agreement, 39 studies were included in this literature review.

### 4.1. Word: A Preferred Treatment Target in Self-Administered Therapies

The review highlights a predominant focus on word-level interventions with 24 of the 39 studies reviewed addressing this target. However, other specific symptoms are also targeted, including sentence processing/production, reading comprehension, and narrative production. Additionally, some studies adopted a more holistic approach by addressing multiple domains, including language and cognitive functions.

Targeting the single word is a common outcome in standard care, where word production is a primary focus in rehabilitation [83], as word-finding difficulties are among the most prevalent and sometimes the only visible symptoms of aphasia. In addition, this emphasis on word production may reflect the relative ease of implementing word-level treatments in a self-administered setting, due to the possibility to design predictive cues and feedback. Implementing effective feedback for more complex tasks, such as narrative or sentence-level tasks, which are well-tailored to the production of the PWA, may be more challenging, making them more difficult to implement in a self-administered setting. The papers included in this review that addressed sentence-level or narrative production focused predominantly on script training. This approach avoids the need for tailored feedback and instead relied on a gradual reduction in cues. However, advances in automatic speech recognition technologies allow for immediate and personalized feedback and have the potential to improve the implementation of self-administered therapies, extending beyond word production.

Among the word production treatments reviewed in this article, the majority primarily focus on noun retrieval, with only a few studies addressing verbs, proper nouns, or adjectives. This aligns with existing literature on anomia treatment, which most often targets concrete nouns due to their high imageability, with few studies exploring less concrete words (such as adjectives, adverbs, or abstract concepts) [84]. In addition, the prevalence of self-administered therapies focusing on nouns may be attributed to the greater ease of designing tasks with predictive feedback for concrete words compared to less tangible words, such as many verbs or adjectives.

### 4.2. Effectiveness of Self-Administered Therapies: Beyond Trained Items

The findings of our review confirm the effectiveness of computerized or smart-tablet-based self-administered therapies in improving word production of trained items with good maintenance over time but rare generalization and transfer. These results are consistent with a previous systematic review on technology-based therapies for anomia [28]. While transfer to connected speech is possible after anomia treatment, the functional benefit of targeting words relevant to PWA appears evident. However, most studies rely on generic vocabulary databases, with less focus on functionally relevant words for PWA. In clinical practice, personalized materials can be challenging to provide, due to time constraints. However, technology can facilitate the selection of functional items, for instance, by involving patients or families in capturing photos of meaningful objects [12].

For the first time, this review also examines the efficacy of these treatments beyond word production, extending to sentence processing and/or production, reading comprehension, and narrative production. In addition to demonstrating the effectiveness of these therapies for trained items and their maintenance over time, the findings provide promising evidence of transfer. The majority of these studies measured and demonstrated transfer to connected speech or perceived improvements reported by PWAs and/or their significant others. For instance, following self-administered script training, PWAs showed significant improvements in the accuracy of script production during conversations [55], and family members reported increased spontaneity and initiation of verbal interactions beyond the trained scripts [57,58]. In another study on sentence-level intervention [59], narrative production improved significantly, with longer utterances and a higher proportion of complex sentences. Additionally, the study specifically targeting narrative production revealed a significant increase in both the number and percentage of Correct Information Units during narrative tasks [64]. These findings are consistent with existing literature suggesting that transfer is more prevalent in therapies emphasizing verbs and sentences [85]. Interestingly, despite the absence of a real interlocutor during self-administered sessions, factors such as greater intensity, autonomy, and accessibility may contribute to successful transfer.

### 4.3. Clinician Involvement in Self-Administered Therapies: A Parameter Not to Be Ignored

In the majority of the studies reviewed, it is mentioned that introductory and training sessions were provided prior to the self-administered therapy. Although this was not specified in all studies, it can be assumed that explanations to PWA regarding the therapy process and the use of the computerized system are necessary before therapy to effectively carry out self-administered therapy [86].

During self-administered therapy, most of the included studies proposed weekly clinician interventions—either in person or remotely—to observe and/or monitor progress without providing any cues or reinforcement. This clinician involvement was particularly necessary when the applications used were unable to adjust the treatment plan or item difficulty based on the performance of PWAs, or to ensure compliance with the treatment protocol. As technology advances and self-learning applications become more sophisticated, the clinician’s monitoring workload is likely to decrease. Some rehabilitation programs have already created a face-to-face treatment environment through virtual reality, where virtual SLP displays facial movements modeling the articulators (Web-ORLA [63]; AphasiaScripts [55,56,57,58]) or provides instructions, explanations, and modeled target sentences (Sentactics; [59]). The emerging use of virtual reality, with immersive or semi-immersive environments that simulate real-life contexts, enhances the ecological validity of language rehabilitation [87,88].

Moreover, regular contact with a clinician has been shown to increase patient adherence [86]. In their study on adherence to self-administered computer therapy, Harrison and colleagues [89] demonstrated that the time spent by the SLP on monitoring and adjusting the software for participants was directly associated with greater adherence to therapy. Regular contact can also be provided by trained volunteers. In the included study by Palmer and colleagues [39], three of the four participants who did not practice with the recommended frequency lacked support from volunteers. Less clinician contact can lead to a decline in patient motivation, as clinicians often provide essential reinforcement and encouragement [50]. These results highlight the value of regular contact with a clinician or volunteer. Such contact can be facilitated through various means, including clinic visits (e.g., [62]), home visits (e.g., [35]), video conferences (e.g., [36]), or phone calls (e.g., [39]).

A few of the studies reviewed offer therapy sessions that actively involve an SLP. In their study, Gallée and colleagues [48] examined the influence of clinician implication and demonstrated that weekly guidance from an SLP, resulted in greater engagement and greater improvement on standardized language assessments compared to independent, unsupervised therapy.

In any case, the role of the SLP remains essential in self-administered therapy. While self-administered settings offer greater intensity, autonomy, and accessibility, they may lack the personalized feedback, dynamic adjustments, and interactive support that are an integral part of therapist-led interventions. In addition, it cannot substitute for clinical expertise in areas such as selecting functionally relevant therapy targets, tailoring cues to the patient’s individual profile, monitoring progress, and ensuring that treatment goals are achieved.

### 4.4. Limitations

It is important to acknowledge that this review presents a number of limitations and conclusions that should be interpreted in the context of these limitations. Firstly, the fact that the majority of treatments reviewed in this study resulted in improvements could be linked to publication bias, as studies without positive outcomes are typically more difficult to publish. Secondly, there is a possibility that some relevant papers may have been omitted, partly due to the lack of consensus on the terminology used to define self-administered SLT. Thirdly, the focus on a specific population—chronic PWA—limits the generalizability of the results to the acute stage. It is hypothesized, however, that computer- or smart-tablet-based therapy could also be effective in acute cases, where recovery is often more significant and rapid due to the combined effects of spontaneous recovery and therapeutic intervention. Nonetheless, we believe that the present work provides valuable insight for clinicians.

## 5. Conclusions

The results of this systematic review confirm that self-administered computer or smart tablet-based treatments, when guided by a SLP, are an effective rehabilitation approach for individuals with chronic post-stroke aphasia.

The large number of studies focusing on anomia have demonstrated positive outcomes for trained items with good maintenance over time but rare generalization and transfer. While fewer studies have explored self-administered therapies targeting symptoms beyond word production—such as sentence processing and/or production, reading, or narrative production—thus far, the findings are promising. Particularly, these studies highlight encouraging transfer effects to connected speech or perceived change by PWA and/or a significant other, which is often challenging to achieve in traditional SLT [85,90].

Although constant clinician supervision is not necessary, guidance sessions can significantly enhance therapy outcomes. The expert role of the SLP remains essential for defining objectives, supervising therapy, and ensuring therapeutic progress.

Finally, it is important to consider some of the challenges associated with self-administered technology-based treatments: high costs and limited access to tablets and applications, potential difficulties in using tablets for PWA, and cognitive impairments that may affect the ability to use technology independently [31].

## Figures and Tables

**Table 1:** Overview of the eligibility criteria used for the selection of articles.

Eligibility Criteria	Inclusion Criteria	Exclusion Criteria
Population	People with a diagnosis of chronic aphasia following stroke (>6 months post-onset)	People with other pathologies (e.g., neurodegenerative disease, craniocerebral trauma, tumors)
Rehabilitation	Self-administered design (frequency of self-administered sessions higher than sessions in the presence of the clinician) Intervention targeting the restoration of a language disorder Computer or smart tablet-based	Augmentative and alternative communication Surgical interventions Neuromodulatory rehabilitation Pharmacological treatments
Types of studies	Peer-reviewed publication Randomized controlled trials Non-randomized controlled group trials Single subject designs Crossover design study	(Systematic) reviews or meta-analysis without original data Research protocols Preliminary Results Conference reports Book chapters

**Table 3:** Clinician involvement with specification of number or intensity of sessions.

Study	Prior to Treatment		During Treatment	
	Additional SLP-Led Sessions	Introduction and Training	Additional SLP-Led Sessions	Observation and/or Monitoring
Pedersen et al., 2001 [51]		NR		
Linebarger et al., 2001 [60]		NR		1×/week
Fink et al., 2002 [49]				1×/week
Laganaro et al., 2003 [46]	2 weeks	1–2 sessions		
Mortley et al., 2004 [38]		1 session		< 1×/month
Choe et al., 2007 [47]		1 session	2×/week	
Ramsberger and Marie, 2007 [50]		2 sessions		
Cherney and Halper, 2008 [57]				1×/week
Fridriksson et al., 2009 [45]		1 session		
Manheim et al., 2009 [58]		5 sessions		1×/week
Choe et al., 2010 [42]		NR	2×/week	
Thompson et al., 2010 [59]				all sessions
Cherney, 2010 [62]				all sessions
Choe and Stanton, 2011 [53]		1 session		
Ball et al., 2011 [33]			1×/week	
Mason et al., 2011 [54]	4 weeks			
Palmer et al., 2012 [39]		NR		1–4×/month
Herbert et al., 2012 [52]		NR		
Kurland et al., 2014 [36]	2 weeks	NR		1×/week
Cherney et al., 2014 [56]				1×/week
Kiran et al., 2014 [68]				1×/week
Des Roches et al., 2015 [67]		NR		1×/week
Lavoie et al., 2016 [34]		2 sessions		
Routhier et al., 2016 [35]		2 sessions		
Choi et al., 2016 [66]				1×/day
Stark and Warburton, 2016 [65]		1 session		
Duncan and Small, 2017 [64]				1×/week
Kurland et al., 2018 [37]	2 weeks	2–3 sessions		1×/week
Lavoie et al., 2019 [12]		1–2 sessions		1st session
Palmer et al., 2019 [14]				NR
Cherney et al., 2019 [55]				all sessions
Gallée et al., 2020 [48]		1 session	1×/week (1/2 group)	
Braley et al., 2021 [10]				2×/week
Cherney et al., 2021 [63]		2 sessions		1×/week
Quique et al., 2022 [44]		4 sessions	1×/week	
Heide et al., 2023 [41]				2×/week
Choe et al., 2023 [43]		1 session		
Trevittaya and Chinchai, 2023 [40]				
Hickin et al., 2023 [61]		1 session	1×/week	
	4/39 *	24/39	6/39	21/39

Note. * These fractions refer to the number of studies proposing the type of clinician involvement mentioned in the column compared to the total number of studies included, highlighted in gray when not applicable. NR, number or intensity of sessions is not reported.

## Data Availability

Not applicable.

## Abbreviations

ORLA: Oral Reading for Language in Aphasia. PRISMA: Preferred Reporting Items for Systematic Reviews and Meta-Analyses. PWA: People With Aphasia. SLP: Speech and Language Pathologist. SLT: Speech and Language Therapy.
